# Preprocessing of gene expression data by optimally robust estimators

**DOI:** 10.1186/1471-2105-11-583

**Published:** 2010-11-30

**Authors:** Matthias Kohl, Hans-Peter Deigner

**Affiliations:** 1 Department of Mechanical and Process Engineering, Furtwangen University, Jakob-Kienzle-Str. 17, 78054 Villingen-Schwenningen, Germany; 2 Fraunhofer Institute for Cell Therapy and Immunology, AG EXIM, Schillingallee 68, 18057 Rostock, Germany

## Abstract

**Background:**

The preprocessing of gene expression data obtained from several platforms routinely includes the aggregation of multiple raw signal intensities to one expression value. Examples are the computation of a single expression measure based on the perfect match (PM) and mismatch (MM) probes for the Affymetrix technology, the summarization of bead level values to bead summary values for the Illumina technology or the aggregation of replicated measurements in the case of other technologies including real-time quantitative polymerase chain reaction (RT-qPCR) platforms. The summarization of technical replicates is also performed in other "-omics" disciplines like proteomics or metabolomics.

Preprocessing methods like MAS 5.0, Illumina's default summarization method, RMA, or VSN show that the use of robust estimators is widely accepted in gene expression analysis. However, the selection of robust methods seems to be mainly driven by their high breakdown point and not by efficiency.

**Results:**

We describe how optimally robust radius-minimax (rmx) estimators, i.e. estimators that minimize an asymptotic maximum risk on shrinking neighborhoods about an ideal model, can be used for the aggregation of multiple raw signal intensities to one expression value for Affymetrix and Illumina data. With regard to the Affymetrix data, we have implemented an algorithm which is a variant of MAS 5.0.

Using datasets from the literature and Monte-Carlo simulations we provide some reasoning for assuming approximate log-normal distributions of the raw signal intensities by means of the Kolmogorov distance, at least for the discussed datasets, and compare the results of our preprocessing algorithms with the results of Affymetrix's MAS 5.0 and Illumina's default method.

The numerical results indicate that when using rmx estimators an accuracy improvement of about 10-20% is obtained compared to Affymetrix's MAS 5.0 and about 1-5% compared to Illumina's default method. The improvement is also visible in the analysis of technical replicates where the reproducibility of the values (in terms of Pearson and Spearman correlation) is increased for all Affymetrix and almost all Illumina examples considered. Our algorithms are implemented in the R package named RobLoxBioC which is publicly available via CRAN, The Comprehensive R Archive Network (http://cran.r-project.org/web/packages/RobLoxBioC/).

**Conclusions:**

Optimally robust rmx estimators have a high breakdown point and are computationally feasible. They can lead to a considerable gain in efficiency for well-established bioinformatics procedures and thus, can increase the reproducibility and power of subsequent statistical analysis.

## Background

Affymetrix microarrays consist of a number of probe cells, each probe cell containing a unique probe. There are two types of probes, perfect match (PM) and mismatch (MM) occurring as pairs. The sequences for PM and MM are almost identical. The difference consists of a single base change in the middle of the PM probe sequence to the Watson-Crick complement for the MM probe sequence. A series of such probe pairs forms a probe set which represents a transcript [1].

Hence, it is part of the preprocessing of Affymetrix arrays to compute a single expression value for the different probe sets. One of the most popular algorithms for this purpose is MAS 5.0, developed by Affymetrix [1]. It is the algorithm that, for instance, was most frequently applied within the framework of phase II of the microarray quality control (MAQC) project [2].

MAS 5.0 uses PM and Ideal Match (IM) to compute the expression values where, for probe set *i *and probe pair *j*,

(1) $${\text{IM}}_{i,j}=\{\begin{array}{cc} {\text{MM}}_{i,j}, & {\text{MM}}_{i,j}<{\text{PM}}_{i,j}\\ 2^{-{\text{SB}}_{i}}{\text{PM}}_{i,j}, & {\text{MM}}_{i,j}\geq {\text{PM}}_{i,j}\\ & \text{and}\;{\text{SB}}_{\text{i}}>\tau_{1}\\ 2^{-\frac{\tau_{1}\tau_{2}}{\tau_{1}+\tau_{2}-\text{SB}}}{\text{PM}}_{i,j}, & {\text{MM}}_{i,j}\geq {\text{PM}}_{i,j}\\ & {\text{and SB}}_{\text{i}}\leq \tau_{1} \end{array}$$

with default values *τ*_1 _= 0.03 (contrast tau) and *τ *_2 _= 10 (scale tau). The specific background (SB*_i_*) is determined using Tukey's biweight one-step estimator (*T*_bi_) where Affymetrix's version of Tukey's biweight disregards all observations outside of median ±5 median absolute deviation (MAD) (i.e. unstandardized MAD) leading to a very robust estimator:

(2) $${\text{SB}}_{i}=T_{\text{bi}}(\log_{2}({\text{PM}}_{i,j}/{\text{MM}}_{i,j}),\;j=1,\;\ldots ,\;n_{i})$$

Then, the signal log value for each probe set is obtained via

(3) $$\text{SigLogVa}1_{i}=T_{\text{bi}}({\text{PV}}_{i,j},\;j=1,\;\ldots ,\;n_{i})$$

with probe value PV*_i, j _*= log_2_(*V_i, j_*) and *V_i, j _*= max{PM*_i, j _*- IM*_i, j_*, *δ*}. The constant *δ *with default value *δ *= 2^-20 ^is introduced for numerical stability.

However, as table three in Section 2.6 of Hampel et al. (1986) [3] suggests there are more efficient robust estimators than Tukey's biweight, e.g. the Tanh-estimator. In addition, Table eight.five in Section 8.7 of Kohl (2005) [4] shows that in the infinitesimal robust setup for normal location and scale one may lose up to 54.9% asymptotic efficiency if one uses Tukey's biweight in combination with MAD (TuMad) instead of the optimally robust estimator. Consequently, we implemented an algorithm along the l ines of MAS 5.0 where we substituted the Tukey biweight one-step location estimator by an in infinitesimally robust radius-minimax (rmx) *k*-step (*k *≥ 1) location *and *scale estimator [5].

Illumina BeadArrays are based on 3-micron silica beads that are randomly positioned on fiber optic bundles or planar silica slides. Each bead is covered with hundreds of thousands of copies of a specific oligonucleotide sequence assigning the bead to a certain bead type, where each bead type is represented in high redundancy with more than 30 replicates on average. The intensity values of the single beads are called *bead level data*. It is part of the preprocessing to aggregate the bead level data to so-called *bead summary data *leading to a single expression value for each bead type. In this paper we only consider data from single-channel gene expression BeadArrays. Besides that, BeadArrays can also be used for SNP genotyping, methylation profiling, and copy number variation analysis [6].

In Illumina's proprietary BeadStudio Software an outlier rejection method (median ±3 × MAD) combined with mean and standard deviation is used to aggregate the bead level data to bead summary data. The MAD in this setup is standardized by 1.4826 to be consistent at the normal model. That is, the location part of Illumina's estimator is a Huber-type skipped mean and is very close to estimator X42 of Hampel (1985) [7], which uses 3.03 × MAD. Quoting Hampel et al. (1986) [3], p. 69, the X42 estimator is "frequently quite reasonable, according to present preliminary knowledge". However, Monte-Carlo studies show that there may be an efficiency loss of 5-25% compared to more sophisticated robust estimators [7]. Hence, we implemented an algorithm which uses rmx *k*-step estimators for summarizing bead level data. The goal of this paper is to demonstrate that the application of optimally robust rmx estimators can lead to a considerable efficiency gain and increased reproducibility for well-established preprocessing algorithms. First, we argue for normal location and scale as the ideal model, at least for the log-transformed values of some publicly available Affymetrix and Illumina data sets, using minimum Kolmogorov distance. Second, we use Monte-Carlo simulations and those publicly available datasets to compare MAS 5.0 and Illumina's default method with our modified algorithms based on rmx estimators. The proposed preprocessing algorithms are implemented in the R package RobLoxBioC[8,9]. A brief overview of infinitesimal robustness is given in the Methods section at the end of the manuscript.

## Results and Discussion

### Affymetrix Data

**Figure 1:**
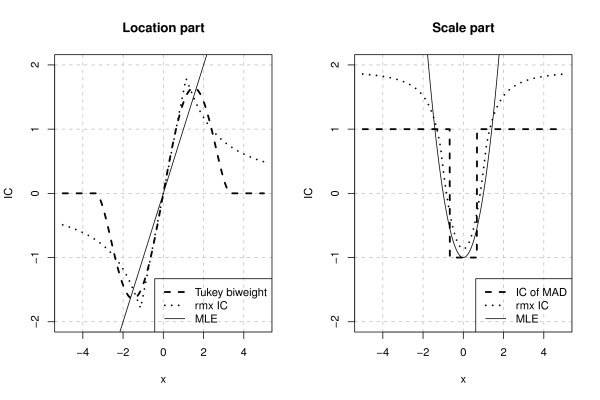
**Location and scale ICs**. Comparison of location and scale ICs for Tukey's biweight, the rmx estimator (rmx IC) and the maximum likelihood estimator (MLE).

We replace Tukey's biweight which only provides a location estimate by an rmx estimator for normal location and scale $N(\mu ,\sigma^{\text{2}})$. The Tukey biweight and the rmx estimator are constructed as one-step and *k*-step estimates based on median and MAD, respectively. As both estimators are so called asymptotically linear (AL) estimators, a straightforward way to compare these estimators is to observe the corresponding influence curves/functions (ICs) displayed in Figure 1.

In both cases, the location part of the IC is redescending. In contrast to Tukey's biweight rejecting observations more than about 3.35 (standardized) MAD times away from the median, the rmx estimator only downweights large observations. Moreover, the plot shows that Tukey's biweight is mostly affected by undiscoverable (very likely to occur in the normal model) gross errors located around 1.51 (standardized) MAD-times away from the median where the IC of Tukey's biweight (and the MAD) is maximal, whereas the rmx estimator is mostly deflected by large observations where the Euclidean length of the location and scale IC is maximal.

However, for applying the rmx estimator for normal location and scale, one first should check if it is plausible to assume normal location and scale as the ideal model for the *M_i, j _*:= log_2_(PM*_i, j_*/MM*_i, j_*) values. As we can not test for *approximate *normality (there is no such statistical test), we use the minimum Kolmogorov distance for this purpose. That is, we minimize, in *μ *∈ ℝ and *σ *∈ (0, ∞),

(4) $$d_{\kappa }(N(\mu ,\;\sigma^{2}),{\hat{F}}_{M_{i}})=\underset{x\in \mathbb{R}}{\sup}|\Phi_{\mu ,\sigma }(x)-{\hat{F}}_{M_{i}}(x)|$$

where Φ *_μ,σ _*is the cumulative distribution function of $N(\mu ,\sigma^{\text{2}})$ and ${\hat{F}}_{M_{i}}$ is the empirical distribution function of the sample *M*_*i, j*_,..., *M*_*i, ni*_. Working with a right-continuous empirical distribution function the above supremum is equal to

(5) $$\underset{j=1,\ldots ,n_{i}}{\max}\left\{\Phi_{\mu ,\sigma }\left(M_{i,(j)}\right)-\frac{j-1}{n_{i}},\;\frac{j}{n_{i}}-\Phi_{\mu ,\sigma }\left(M_{i,(j)}\right)\right\}$$

where *M*_*i*,(1), _..., *M*_*i*,(*ni*) _is the increasingly sorted sample. In particular, the minimal possible Kolmogorov distance for sample size *n *is (2*n*)^-1^.

Of course, it would be possible to use some other distance (e.g. Cramér von Mises) or the test statistic of some test for normality for this purpose. However, we decided to use the Kolmogorov distance since there is a certain connection between Kolmogorov neighborhoods and the gross-error model in infinitesimal robustness (see Rieder (1994), Lemma 4.2.8 and Proposition 5.3.3 [10]) and the Kolmogorov distance can be computed efficiently via equation (5). Nevertheless, the computations take more than 100 minutes for the HGU95A dataset and more than 130 minutes for the HGU133A dataset, which consist of 59 and 42 GeneChips, respectively, using function KolmogorovMinDist of our package RobLoxBioC on an Intel P9500 (64 bit Linux, 8 GByte RAM). For more details on these Latin square spike-in datasets we refer to Cope et al. (2004) [11] and Irizarry et al. (2006) [12].

**Figure 2:**
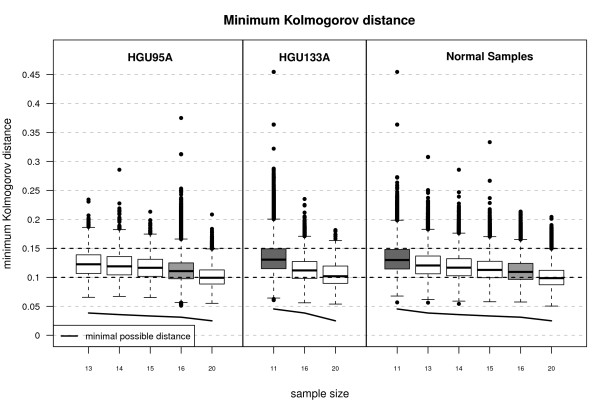
**Minimum Kolmogorov distance for Affymetrix data**. Minimum Kolmogorov distances for HGU95A and HGU133A datasets as well as for normal (pseudo) random samples (50000 Monte-Carlo replications). The grey boxes indicate the mode of the number of probes in a probe-set. The figure indicates that the considered Affymetrix data is in good agreement with the normal location and scale model.

Table 1 shows the number of probe sets per number of probe level pairs for the HGU95A and HGU133A GeneChips. Figure 2 displays the minimum Kolmogorov distances for the HGU95A and HGU133A Latin square datasets as well as for normal random samples (50000 Monte-Carlo replications for each sample size) where we selected only those probe level pairs with a considerable number of probe sets. In Table 2 we recorded the differences of the medians of the minimum Kolmogorov distances between the Latin square datasets and corresponding normal random samples. The results for 95% and 99% quantiles are very similar. Based on these results it is very reasonable to assume normal location and scale as the ideal model for *M_i, j _*expecting only minor deviations from normality.

**Table 1:** Number of probe sets

No. of PP	6	7	8	9	10	11	12	13	14	15	16	20	69
**HGU95A**	8	3	4	4	1	4	11	53	45	40	12386	66	1

**HGU133A**	0	0	1	0	1	21765	0	4	4	2	482	40	1

Number of probe sets per number of probe level pairs (No. of PP) for HGU95A and HGU133A GeneChips.

**Table 2:** Minimum Kolmogorov distances for Affymetrix data

No. of PP	6	7	8	9	10	11	12
**HGU95A**	0.002	0.0012	0.0157	-0.0009	-0.0146	0.0267	0.0040

**HGU133A**			-0.0098		-0.0079	0.0007	

							

**No. of PP**	**13**	**14**	**15**	**16**	**20**	**69**	

**HGU95A**	0.0020	0.0022	0.0036	0.0014	0.0006	0.0156	

**HGU133A**	0.0076	0.0033	0.0117	0.0026	0.0032	0.0148	

Differences of the medians of the minimum Kolmogorov distances between the Latin square datasets and the normal random samples for each number of probe level pairs (No. of PP).

To get a rough estimate of the corresponding size of the contamination neighborhood (i.e. Tukey's gross-error model [13]), which is required for selecting an appropriate rmx estimator, we use the following heuristics: for Kolmogorov (*U_k_*), total variation (*U_v_*) and contamination neighborhoods (*U_c_*) of size *s *∈ (0,1) it holds

(6) $$U_{c}(s)\subset U_{v}(s)\subset U_{\kappa }(s)$$

In addition, at least in the one-dimensional case and under symmetry, the optimally robust ICs for *U_c_*(2*s*) and *U_v_*(*s*) coincide. Moreover, if the optimally robust IC is monotone, then it is also the solution for *U_k _*(*s*) [10]. Based on these considerations we multiply the median difference between the observed and the simulated Kolmogorov distance by two, leading us to a neighborhood size *s *∈ [0, 0.05].

We use the corresponding rmx 3-step estimator instead of Tukey's biweight to compute the specific background values SB*_i _*and the signal log values SigLogVal*_i_*. Asymptotically (i.e. classical first-order asymptotics) speaking it makes no difference which *k *we choose to construct the rmx estimator and differences only occur if one takes a look at higher-order asymptotics as shown by unpublished results of P. Ruckdeschel. However, to date, there are no finite-sample results indicating an optimal choice for *k *if there is any. The use of three steps is motivated by the observation that in all situations we considered so far, the estimates were stable and did not change very much after the third iteration.

The results in Section 8.7 of Kohl (2005) [4] show that, in the infinitesimal robust setup and for known contamination radius, the optimally robust AL estimators clearly outperform the TuMad estimator for the estimation of normal location and scale with respect to the asymptotic maximum MSE, where the maximum efficiency loss is 54.9%. Moreover, the results of the following Monte-Carlo study, which is in the spirit of the Princeton robustness study [14], indicate that this is also true for the rmx estimator in the case of an unknown neighborhood radius and finite-sample size. Due to the finite-sample size and the shrinkage of the neighborhoods, we use a finite-sample correction of the neighborhood radius determined by a large simulation study. The finite-sample correction leads to a larger neighborhood radius; i.e., to a more conservative estimation procedure. It can be computed with function finiteSampleCorrection of the R package RobLox[15].

For the simulations we chose a sample size *n *= 11 as most of the probes sets have this number of probe pairs on HGU133A GeneChips (cf. Table 1) and performed *M *= 10^5 ^Monte-Carlo replications. As the ideal model we used N(0,1) which is no restriction due to equivariance of the location and scale model. As contaminating (gross errors generating) distributions we employed:

• N(0,9), *t*_3 _and Cauchy(0, 1) leading to an increased variance

• N(3,1) and N(10,1)causing a positive bias

• Dirac measures at 1.51 (*D*_1.51_) and 1000 (*D*_1000_), which are approximations for the least favorable contaminations (i.e. leading to maximum risk) for the Tukey and the rmx estimator, respectively.

We selected *s *∈ {0.01, 0.02, 0.04} as sizes of the gross error models. The results for other contaminating distributions or amounts of gross errors can easily be computed with function AffySimStudy of our R package RobLoxBioC.

Since there is no estimator yielding reliable results if there are 50% or more gross errors, we wanted to admit only random samples with less than 50% contamination. The probability for rejecting a sample is ≤ exp{-2*n*(0.5 - *s*)^2^} by Theorem 2 of Hoeffding (1963) [16]; i.e., decays exponentially in the sample size *n*. At *n *= 11 and *s *∈ {0.01, 0.02, 0.04} a replacement of a sample is necessary with probability 4.4 · 10^-10^, 2.7 · 10^-8 ^and 1.6 · 10^-6^, respectively. Hence, unsurprisingly, there was no single sample that had to be replaced in our Monte-Carlo study.

The results in Table 3 show that the rmx location estimate in all situations considered has a smaller empirical MSE than Tukey's biweight. The efficiency loss of Tukey's biweight in nearly all situations is about 15-20%.

**Table 3:** Simulation study: Tukey's biweight versus rmx estimator

	increased variance			positive bias		least favorable	
	**N(0,9)**	***t*_3_**	**Cauchy**	**N(3,1)**	**N(10,1)**	***D***_**1**.**51**_	***D***_**1000**_

***s *= 0.01**							

**Location**.							

MLE	1.802	1.022	300.548	1.100	2.103	1.017	> 10^4^

median	1.554	1.516	1.522	1.551	1.551	1.551	1.551

biweight	1.325	1.316	1.317	1.343	1.319	1.346	1.318

rmx	1.109	1.096	1.098	1.131	1.105	1.117	1.097

**Location and scale:**							

MLE	6.987	1.638	3293.342	1.745	7.616	1.541	> 10^5^

median & MAD	2.919	2.864	2.876	2.930	2.936	2.908	2.936

TuMAD	2.700	2.663	2.671	2.723	2.704	2.703	2.704

rmx	1.811	1.736	1.752	1.804	1.853	1.742	1.855

***s *= 0.02**							

**Location:**							

MLE	2.575	1.045	405.083	1.216	3.387	1.037	> 10^4^

median	1.575	1.519	1.528	1.593	1.595	1.595	1.595

biweight	1.339	1.318	1.322	1.380	1.327	1.386	1.326

rmx	1.131	1.100	1.106	1.184	1.135	1.149	1.105

**Location and scale:**							

MLE	12.311	1.756	4439.072	1.975	13.890	1.548	> 10^5^

median & MAD	2.986	2.870	2.892	3.014	3.032	2.963	3.032

TuMAD	2.749	2.669	2.685	2.802	2.764	2.755	2.764

rmx	1.931	1.744	1.777	1.907	2.071	1.761	2.093

***s *= 0.04**							

**Location:**							

MLE	4.192	1.096	> 10^4^	1.497	6.557	1.125	> 10^4^

median	1.643	1.526	1.546	1.696	1.701	1.627	1.701

biweight	1.368	1.322	1.331	1.476	1.344	1.415	1.338

rmx	1.182	1.106	1.119	1.326	1.285	1.197	1.116

**Location and scale:**							

MLE	23.633	2.063	> 10^5^	2.513	27.267	1.690	> 10^5^

median & MAD	3.151	2.883	2.930	3.232	3.301	3.030	3.301

TuMAD	2.875	2.680	2.716	3.012	2.944	2.818	2.938

rmx	2.305	1.759	1.828	2.192	2.984	1.858	3.289

Empirical MSE (multiplied by *n*) for normal location and scale estimators at sample size *n *= 11 (10^5 ^Monte-Carlo replications) and contamination *s *∈ {0.01, 0.02, 0.04}.

Next, we present some results demonstrating the accuracy of the two procedures for the HGU95A and HGU133A Latin square datasets. For the computations we also used the rmx 3-step estimator for *s *∈ [0, 0.05] which is implemented as default in function RobLoxBioC of our R package RobLoxBioC. The results for the MAS 5.0 with Tukey's biweight were determined with function mas5 of Bioconductor package affy[17,18]. In addition to the availability of different robust estimators, the implementation in RobLoxBioC is more efficient. The normalization using RobLoxBioC on an Intel P9500 (64 bit Linux, 8 GByte RAM) requires about 1 minute in contrast to about 9 minutes for mas5.

**Figure 3:**
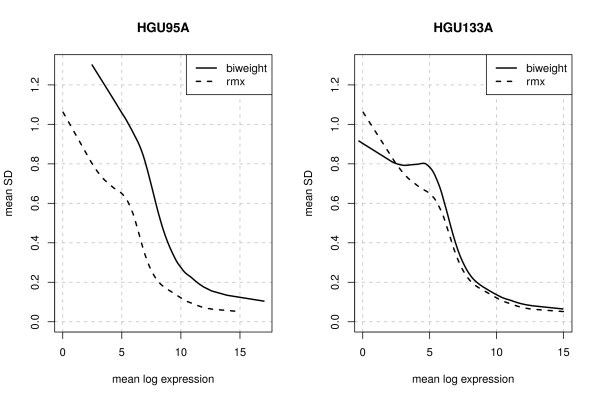
**Tukey's biweight versus rmx estimator**. Mean standard deviation (SD) versus mean log expression for Tukey's biweight and the rmx estimator for *s *∈ [0,0.05]. The curves were determined by smoothing the resulting scatterplots which include SDs and mean log expressions for each gene not spiked-in. As the variability in terms of mean SD for the rmx estimator is clearly smaller especially for HGU95A, these plots indicate an improvement of the accuracy of MAS 5.0 by using the rmx estimator instead of Tukey's biweight.

In Figure 3 analogously to Figure 2 in Cope et al. (2004) [11], the mean standard deviations (SDs) are plotted against the mean log-expression values for the two datasets. The curves were determined by smoothing the resulting scatterplots, which include SDs and mean log expressions for each gene not spiked-in. These plots indicate an improvement of the accuracy of MAS 5.0 when using the rmx estimator instead of Tukey's biweight as the variability in terms of mean SD for the rmx estimator is clearly smaller especially for HGU95A. Some quantiles of the computed SD values are given in Table 4. The results for the log fold-changes observed for non-differentially expressed genes (null log-fc) -i.e., genes not spiked-in - confirm these results; see Table 4. Overall we expect that using the rmx estimator increases the accuracy of MAS 5.0 by 10-20%.

**Table 4:** Accuracy measures: Tukey's biweight versus rmx estimator

	HGU95A		HGU133A	
**Estimator**	**rmx**	**biweight**	**rmx**	**biweight**

25% SD	0.285	0.298	0.136	0.153

median SD	0.535	0.595	0.256	0.292

75% SD	0.844	0.918	0.498	0.639

99% SD	1.391	1.524	1.266	1.381

null log-fc IQR	0.789	0.842	0.400	0.468

null log-fc 99%	3.073	3.356	2.504	2.866

null log-fc 99.9%	4.098	4.455	3.827	4.214

Accuracy measures of [11] and [12] (smaller is better) for Tukey's biweight and the rmx estimator for *s *∈ [0,0.05].

The comparisons of the two robust procedures were performed with the Bioconductor package affycomp[19]. The full assessments of Cope et al. (2004) [11] and Irizarry et al. (2006) [12] can be computed using the R code specified in the file AffymetrixExample.R in the scripts folder of our package RobLoxBioC. The simulation study can be recomputed by the R code given in the file AffymetrixSimStudy.R also provided in the scripts folder.

**Figure 4:**
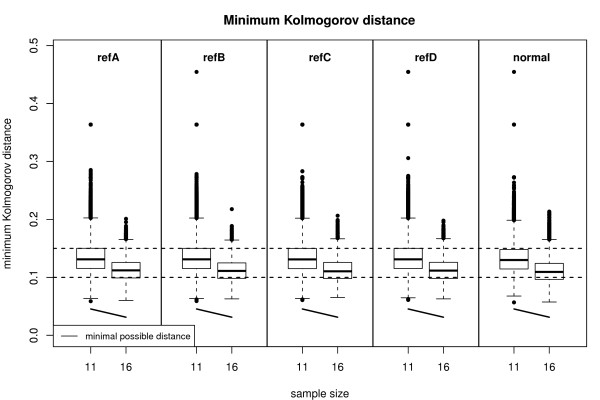
**Minimum Kolmogorov distance for MAQC-I data**. Minimum Kolmogorov distances for randomly selected MAQC-I Affymetrix datasets (refA,..., refD) as well as for normal (pseudo) random samples (50000 Monte-Carlo replications). Only probe-sets with a considerable number of probes are depicted. The boxplots show that the data is in good agreement with the normal location and scale model.

As the following results indicate, the higher accuracy of rmx estimators increases the reproducibility of gene expression analyses. We analyzed a random subset of the MAQC-I study [20] provided by the Bioconductor package MAQCsubsetAFX[21]. Regarding the Affymetrix platform, a total of 120 U133 Plus 2.0 GeneChips have been generated and four different reference RNAs have been used. (A) 100% of Stratagene's Universal Human Reference RNA, (B) 100% of Ambion's Human Brain Reference RNA, (C) 75% of A and 25% of B and (D) 25% of A and 75% of B. Each reference has been repeated five times on six different test sites. The datasets refA,..., refD provided by package MAQCsubsetAFX consist of the data of six randomly chosen U133 Plus 2.0 GeneChips (one for each test site) for each reference RNA. As Figure 4 shows, the assumption of approximate normality is fulfilled. We measured the reproducibility in terms of the Spearman correlation of the normalized data and the Pearson correlation of the log2-transformed normalized data. In all cases the correlation was found to be higher for the rmx estimators. The relative increase is 0.6-1.2% (absolute. 0.006-0.011) in the case of Spearman correlation and 1.2-1.9% (absolute. 0.011-0.017) in the case of Pearson correlation.

The results can be recomputed using the R code specified in the file AffymetrixReproducibility.R in the scripts folder of the package RobLoxBioC.

### Illumina Data

**Figure 5:**
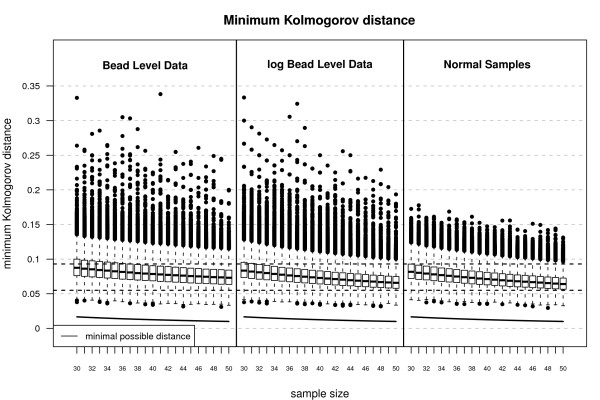
**Minimum Kolmogorov distance for Illumina data**. Minimum Kolmogorov distances for 48 Mouse-6 version 1 BeadChips as well as for normal (pseudo) random samples (50000 replications). The boxplots indicate that the log-transformed bead level data is in good agreement with the normal location and scale model.

**Figure 6:**
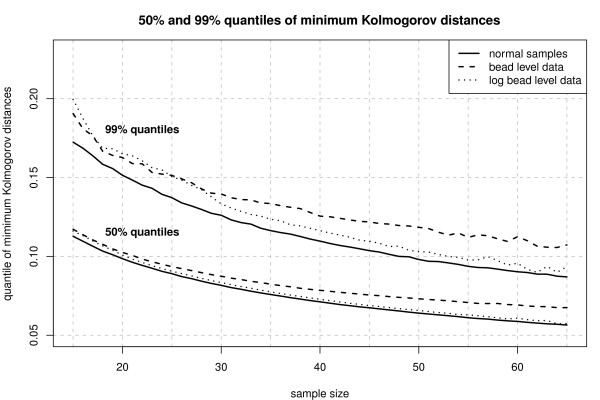
**Quantiles of Minimum Kolmogorov distance for Illumina data**. 50% and 99% quantiles of minimum Kolmogorov distances for 48 Mouse-6 version 1 BeadChips as well as for normal (pseudo) random samples (50000 replications). The plot confirms that the log-transformed bead level data is in good agreement with the normal location and scale model.

Since we intend to apply the rmx estimators for normal location and scale to summarize the bead level data, we first checked whether the normal model is appropriate for these data. We use the spike-in data investigated in Dunning et al. (2008) [22] consisting of eight customized Mouse-6 version 1 BeadChips hybridized with a complex mouse background. Each BeadChip contains six BeadArrays each made up of two strips on the chip surface. In total each of the BeadArrays includes 49283 bead types. The raw bead level values were sharpened and background subtracted [22]. Due to the random positioning of the beads, the number of beads per bead type varies from array to array. In Figure 5 and 6 we have depicted those bead types with 30 to 50 replicates and 15 to 65 replicates, respectively. The results were obtained using function KolmogorovMinDist of the R package RobLoxBioC where the computations took about 9 hours for the spike-in dataset. Both figures indicate that the assumption of normal location and scale as the ideal model is more appropriate for the log-transformed bead level data. Hence, we propose to use the rmx 3-step estimator for normal location and scale and *s *∈ [0, 0.05] in combination with the already mentioned finite-sample correction instead of Illumina's method, which is a Huber-type skipped mean and standard deviation [7], to summarize the log-transformed bead level data. The use of three steps and the choice *s *∈ [0, 0.05] is driven by the same heuristics as in the Affymetrix case.

In a further step we have performed a simulation study using a very similar setup as in the Affymetrix case. Due to the higher redundancy of the Illumina data, we chose a sample size of 30 instead of 11. Moreover, we replaced the Dirac measure at 1.51 by the Dirac measure at 3 (*D*_3_) which is an approximation for the least favorable contamination for Illumina's default method. The results for other contaminations can easily be computed with the function IlluminaSimStudy of the R package RobLoxBioC. As in the Affymetrix setup, we applied the modification that less than 50% of the observations contained in a sample may be contaminated where again no single sample had to be modified. The results in Table 5 show that the two estimators perform similarly with a slight advantage for the rmx estimator. Due to the outlier rejection step included in the Illumina method, it is unsurprising, that it performs especially well if the contaminating distribution puts mass on large values. In contrast, the rmx estimator outperforms the Illumina method in situations where the outliers are less obvious like in the case of *t*_3 _or N(3,1). Furthermore, looking at the maximum empirical risk for the simultaneous estimation of location and scale Illumina's method shows an efficiency loss of about 15% compared to the rmx estimator. In view of these results it is no surprise that Illumina's method performs best in Figure 2 of Dunning et al. (2008) [22] where outliers at 2^16 ^were used and average bias and log2 variance are plotted versus percentage of simulated outliers for several summary methods. Besides that, the approach of Dunning et al. (2008) [22] contains a flaw from a statistical point of view. the original data is contaminated irrespective of the bead type. That is, one gross-error model for the whole dataset was used instead of a gross-error model for each bead type in the dataset. This approach might reflect the way contamination occurs in practice but, already at moderate contamination rates, one obtains many bead types where 50% or more of the bead values are contaminated and consequentially no reliable estimator exists. We postulate that this is the reason why the reported breakdown points are clearly smaller than the "real" breakdown points of the considered estimators (10% trimmed mean and SD: 5% vs. 10%, median and MAD: 30% vs. 50%, Illumina method: 30% vs. 50%).

**Table 5:** Simulation study: Illumina's default method versus rmx estimator

	increased variance			positive bias		least favorable	
	**N(0,9)**	**t_3_**	**Cauchy**	**N(3,1)**	**N(10,1)**	**D_3_**	**D_1000_**

***s *= 0.01**							

**Location**.							

MLE	1.787	1.022	42.929	1.119	2.297	1.110	> 10^4^

median	1.526	1.499	1.505	1.537	1.537	1.537	1.537

Illumina	1.093	1.080	1.086	1.138	1.083	1.177	1.083

rmx	1.095	1.084	1.088	1.119	1.091	1.124	1.086

**Location and scale:**							

MLE	12.482	1.699	1243.738	1.801	13.366	1.704	> 10^5^

median & MAD	2.869	2.818	2.828	2.882	2.885	2.885	2.885

Illumina	1.806	1.786	1.796	1.901	1.780	1.994	1.780

rmx	1.755	1.689	1.705	1.764	1.779	1.778	1.779

***s *= 0.02**							

**Location:**							

MLE	2.563	1.040	756.039	1.282	4.132	1.264	> 10^4^

median	1.553	1.500	1.510	1.583	1.584	1.584	1.584

Illumina	1.110	1.083	1.091	1.214	1.087	1.310	1.087

rmx	1.107	1.085	1.091	1.170	1.099	1.185	1.089

**Location and scale:**							

MLE	23.974	1.880	22605.549	2.190	26.926	1.995	> 10^5^

median & MAD	2.934	2.824	2.842	2.970	2.977	2.977	2.977

Illumina	1.838	1.794	1.810	2.056	1.780	2.276	1.780

rmx	1.862	1.695	1.724	1.886	1.941	1.930	1.941

***s *= 0.04**							

**Location:**							

MLE	4.150	1.080	> 10^4^	1.768	9.587	1.732	> 10^4^

median	1.611	1.502	1.524	1.717	1.720	1.720	1.720

Illumina	1.149	1.096	1.111	1.458	1.102	1.716	1.102

rmx	1.139	1.091	1.102	1.343	1.137	1.413	1.099

**Location and scale:**							

MLE	49.559	2.221	> 10^5^	3.285	59.046	2.879	> 10^6^

median & MAD	3.090	2.833	2.876	3.232	3.256	3.256	3.256

Illumina	1.923	1.816	1.848	2.534	1.787	3.091	1.787

rmx	2.236	1.710	1.781	2.311	2.591	2.485	2.598

Empirical MSE (multiplied by *n*) for normal location and scale estimators at sample size *n *= 11 (10^5 ^Monte-Carlo replications) and contamination *s *∈ {0.01, 0.02, 0.04}.

Next, we report some results representing the accuracy of the two procedures for the spike-in data of Dunning et al. (2008) [22]. For the computations we again used the rmx 3-step estimator for *s *∈ [0, 0.05] which is the default in function RobLoxBioC of the R package RobLoxBioC. The results for Illumina's method were determined with the function createBeadSummaryData of the Bioconductor package beadarray[23]. The computations of the bead summary values take about 100 seconds and about 500 seconds using createBeadSummaryData and RobLoxBioC, respectively. So far, the rmx estimator is implemented in interpreted R code. By switching to compiled code (e.g., C/C++ or FORTRAN) we probably could compete with createBeadSummaryData which is calling C code.

**Figure 7:**
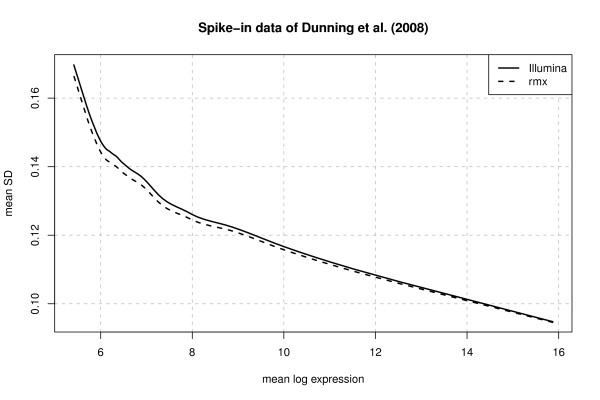
**Illumina's default method versus rmx estimator**. Mean standard deviation (SD) versus mean log expression for Illumina's default method and the rmx estimator for *s *∈ [0,0.05]. The plot indicates a slight improvement of the accuracy by using the rmx estimator instead of Illumina's default method as the mean SD is slightly smaller for the rmx estimator.

For the comparisons we use the approach of Cope et al. (2004) [11] and Irizarry et al. (2006) [12] as in the case of the Affymetrix data. In a first step we plotted the mean SDs against the mean log-expression values for those genes not spiked-in, results are depicted in Figure 7. The plot indicates a slight improvement of the accuracy by using the rmx estimator instead of Illumina's default method as the mean SD is slightly smaller for the rmx estimator. Some quantiles for the SD values are given in Table 6. Secondly, we took a look at the log fold-changes observed for the non-differentially expressed genes (null log-fc). This statistic also confirms the above results; see Table 6. Moreover, we added the results for the other methods implemented in package beadarray which are mean and SD, median and MAD, 5% trimmed mean and SD as well as 5% winsorized mean and SD. The numerical results show that these other methods perform worse than Illumina's default method and the rmx estimator. Overall the rmx estimator performs the best and we expect an increase in accuracy of at least 1-5% by using the rmx estimator instead of the other methods.

**Table 6:** Accuracy measures: Illumina's default method versus rmx estimator

Estimator	rmx	Illumina	Mean	median	5% trim	5% winsorize
25% SD	0.114	0.116	0.125	0.121	0.117	0.122

median SD	0.133	0.135	0.140	0.142	0.133	0.137

75% SD	0.150	0.153	0.157	0.160	0.150	0.153

99% SD	0.308	0.309	0.314	0.310	0.310	0.313

null log-fc IQR	0.186	0.187	0.208	0.208	0.188	0.192

null log-fc 99%	0.362	0.366	0.412	0.408	0.377	0.388

null log-fc 99.9%	0.495	0.504	0.584	0.555	0.525	0.546

Accuracy measures of [11] and [12] (smaller is better) for the rmx estimator for s ∈ [0, 0.05], Illumina's default method as well as mean and SD (mean), median and MAD (median), 5% trimmed mean and SD (5% trim) and 5% winsorized mean and SD (5% winsorize).

The results mentioned can be recomputed via the R code provided in files IlluminaExample.R and IlluminaSimStudy.R which are included in the scripts folder of our R package RobLoxBioC.

As the following results indicate, the higher accuracy of our rmx estimators is reflected in an increased reproducibility of gene expression analyses. We have again used the spike-in data of Dunning et al. (2008) [22] which can be divided into twelve sets each including four technical replicates. For these twelve sets we measured the reproducibility in terms of the Spearman and Pearson correlation of the summarized log2-transformed data, overall leading to 72 pairwise comparisons. In 69 (Spearman correlation) and 66 (Pearson correlation) cases respectively, the correlation was higher for the rmx estimators. As before, the differences between the two procedures were found to be small and remained well below 0.5% in all cases.

The results can be recomputed using the R code given in the file IlluminaReproducibility.R in the scripts folder of our package RobLoxBioC.

## Conclusions

As the variability of the estimation is clearly reduced as well as the reproducibility is increased when we apply rmx estimators for preprocessing, it is reasonable to assume a higher power for subsequent statistical analyses.

In the case of Illumina data the rmx summarization method can be combined with different preprocessing methods that can be applied to bead summary data, e.g. the variance-stabilizing transformation (VST) of Lin et al. (2008) [24].

In the case of Affymetrix data there are several other well-known normalization methods based on parametric models e.g. the robust multi-array average (RMA [25]) or the variance stabilization and calibration (VSN [26]) which can be used. The RMA procedure is based on a linear additive model where one uses median polish [27] for parameter estimation. A replacement of the median polish by a corresponding rmx polish may further improve the algorithm. In the case of VSN a possible modification could consist of replacing the least trimmed sum of squares (LTS) regression [28] by an rmx estimator for regression [4,10]. As the above results and the results in Chapters 7 and 8 in Kohl (2005) [4] indicate, these modifications lead to an increased accuracy. At the same time we can retain the high breakdown point of the already available robust estimators by using the *k*-step construction in combination with bounded rmx ICs [29].

The reported results and the universality of the infinitesimal robustness approach suggest that optimally robust rmx estimators should also be of interest for other bioinformatics applications.

## Methods

### Infinitesimal Robustness

The approach of Huber-Carol (1970) [30], Rieder (1978) [31], Bickel (1981) [32] and Rieder (1980) [33], Rieder (1994) [10] to robust testing and estimation employs shrinking neighborhoods of the parametric model, where the shrinking rate *n*^-1/2^, as the sample size *n *→∞, may be deduced in a testing setup [34]. Due to the shrinkage of the neighborhoods and the asymptotics involved this approach to robustness is called *infinitesimal*. A brief comparison and distinction to the robustness approaches of Huber (1981) [35], Hampel et al. (1986) [3] and Maronna et al. (2006) [36] is given in the Introduction of Kohl et al. (2010) [29].

Denoting by ${ℳ}_{\text{1}}(A)$ the set of all probability measures on some measurable space (Ω,A), one considers a smoothly parameterized model

(7) $$P=\{P_{\theta }|\theta \in \Theta \}\subset {ℳ}_{1}(A)$$

whose parameter space Θ is an open subset of some finite-dimensional ℝ*^k^*, and which is dominated. *dP_θ _*= *p_θ _d μ *(*θ*_∈ _Θ). The smoothness of the model P, at any fixed *θ *∈ Θ is characterized by the requirement of *L*_2 _differentiability (also called differentiability in quadratic mean); see Section 2.3 of [10]. The ℝ*^k^*-valued *L*_2 _derivative is denoted by $\Lambda_{\theta }\in L_{2}^{k}(P_{\theta })$ and its covariance ${ℐ}_{\theta }={\text{E}}_{\theta }\Lambda_{\theta }{\Lambda^{\prime }}_{\theta }$ under *P_θ _*is the Fisher information of P at *θ *which is assumed of full rank *k*. This type of differentiability is for instance implied by continuous differentiability of *p*_*θ *_and continuity of *I_θ _*with respect to *θ *and then $\Lambda_{\theta }=\frac{\partial }{\partial \theta }\log p_{\theta }$ the classical scores; see Lemma A.3 of Hájek (1972) [37].

Given the so-called *ideal model *P one defines asymptotically linear (AL) estimators *S *to be any sequence of estimators *S*_*n*_: Ω^*n *^→ ℝ^*k *^such that

(8) $$\begin{array}{l} \sqrt{n}(S_{n}-\theta )=\frac{1}{\sqrt{n}}\sum_{i=1}^{n}\psi_{\theta }(x_{i})+\text{o}p_{\theta }^{n}(n^{0}) \end{array}$$

for some, necessarily unique, influence curve (IC)*ψ*_*θ *_∈ Ψ(*θ*), where

(9) $$\Psi (\theta )=\{\psi_{\theta }\in L_{2}^{k}(P_{\theta })|{\text{E}}_{\theta }\psi_{\theta }=0,\;{\text{E}}_{\theta }\psi_{\theta }{\Lambda^{\prime }}_{\theta }=I_{k}\}$$

Here we used the stochastic Landau-notation of Pfanzagl (1994) [38], i.e. ${\text{op}}_{\theta }^{n}(n^{0})\to 0$ in product $P_{\theta }^{n}$ probability as *n *→∞, and ℐ*_k _*denotes the k×k identity matrix. For more details we refer to Rieder (1994), Section 4.2 [10].

In infinitesimal robustness, the i.i.d. observations *x*_1_,..., *x_n _*may follow any law *Q *in some shrinking neighborhood about *P_θ_*. In this article, we consider the (convex) contamination neighborhood system $U_{c}(\theta )$ which consists of all contamination neighborhoods, at size 0 ≤ *s *≤ 1,

(10) $$U_{c}(\theta ,\;s)=\{(1-s)P_{\theta }+sQ|Q\in {ℳ}_{1}(A)\}$$

Subsequently, *s *= *s_n _*= *rn*^-1/2 ^for starting radius *r *∈ [0, ∞) and *n *→∞.

Given this setup, the aim is to minimize the asymptotic maximum risk

(11) $$\underset{n\to \infty }{\lim}\underset{Q\in U_{c}(\theta ,rn^{-1/2})}{\sup}\int \ell (n^{1/2}(S_{n}-\theta ))dQ_{n}^{n}$$

with continuous loss function *ℓ: *ℝ*^k ^*→ [0, ∞). Throughout this article we will use square loss *ℓ *(*z*) = |*z*|^2 ^which leads to the (asymptotic maximum) mean squared error MSE*_θ_*(*ψ*_*θ*_, *r*).

To simplify notation, the fixed *θ *will be dropped from notation henceforth.

The optimally robust ψ*, the unique solution to minimize MSE(*ψ*, *r*) among all *ψ *∈ Ψ for given radius *r*, is given in Theorem 5.5.7 of Rieder (1994) [10]: there exist some vector *z *∈ ℝ^*k *^and matrix *A *∈ ℝ^*k *× *k*^, *A *positive definite, such that

(12) $$\psi^{⋆}=A(\Lambda -z)w$$

(13) $$w=\min\{1,\;b|A(\Lambda -z){|}^{-1}\}$$

where

(14) $$r^{2}b=\text{E}{\left(|A(\Lambda -z)|-b\right)}_{+}$$

and

(15) 0=E(Λ−z)w

(16) $$A^{-1}=\text{E}{\left(\Lambda -z)(\Lambda -z\right)}^{\prime }w$$

Conversely, form (12) - (15) suffices for ψ* to be the solution. The minimax solution to more general risks is derived in Ruckdeschel and Rieder (2004) [39].

In applications, the starting radius *r *for the neighborhoods *U*_*c*_(*θ*, *rn*^-1/2^) is unknown or only known to belong to some interval [*r*_lo_, *r*_up_) ⊂ [0, ∞). For this situation Rieder et al. (2008) [5] propose to consider the relative MSE of $\psi_{s}^{⋆}$ at radius *r*, defined as

(17) $$\text{relMSE}(\psi_{s}^{⋆},\;r):=\text{MSE}(\psi_{s}^{⋆},\;r)/\text{MSE}(\psi_{r}^{⋆},\;r)$$

and IC $\psi_{r_{0}}^{⋆}$ which minimizes

(18) $$\underset{r\in [r_{lo},r_{\text{up}})}{\sup}\text{relMSE}(\psi_{s}^{⋆},\;r)$$

among all *s *∈ [*r*_lo_, *r*_up_) is called *radius-minimax *(*rmx*).

Given the rmx IC the corresponding rmx estimator can then be determined via the one-step construction respectively, an iterated one-step - that is, *k*-step (*k *≥ 1) -construction

(19) $$S_{n}^{\left(k\right)}=S_{n}^{\left(k-1\right)}+\frac{1}{n}\sum_{i=1}^{n}\psi_{S_{n}^{\left(k-1\right)},r_{0}}^{⋆}(x_{i})$$

based on a suitable starting estimate $S_{n}^{\left(0\right)}$[29].

The normal location and scale model, i.e. $P_{\theta }=N(\mu ,\;\sigma^{2})\;$ with *θ *= (*μ*, *σ*)', *μ *∈ ℝ, *σ *∈ (0, ∞) forms an *L*_2 _differentiable exponential family. As starting estimator one can use median and median absolute deviation (MAD) as justified by Kohl (2005), Section 2.3.4 [4]. Since the rmx IC in this model is bounded, the breakdown point of the starting estimator, which is 50% for median and MAD, is inherited to the rmx one-step estimator [29].

## Authors' contributions

MK implemented the algorithms, performed the computations and contributed to the manuscript. HPD contributed to the manuscript writing and discussion. Both authors read and approved the final manuscript.
